# Hippocampal formation-inspired global self-localization: quick recovery from the kidnapped robot problem from an egocentric perspective

**DOI:** 10.3389/fncom.2024.1398851

**Published:** 2024-07-18

**Authors:** Takeshi Nakashima, Shunsuke Otake, Akira Taniguchi, Katsuyoshi Maeyama, Lotfi El Hafi, Tadahiro Taniguchi, Hiroshi Yamakawa

**Affiliations:** ^1^Graduate School of Information Science and Engineering, Ritsumeikan University, Osaka, Japan; ^2^Graduate School of Information Science and Technology, Osaka University, Osaka, Japan; ^3^College of Information Science and Engineering, Ritsumeikan University, Osaka, Japan; ^4^Research Organization of Science and Technology, Ritsumeikan University, Shiga, Japan; ^5^The Whole Brain Architecture Initiative, Tokyo, Japan; ^6^School of Engineering, The University of Tokyo, Tokyo, Japan; ^7^RIKEN Center for Advanced Intelligence Project, Tokyo, Japan

**Keywords:** allocentric, brain-inspired AI, egocentric, kidnapped robot problem, Monte Carlo localization, probabilistic generative model, world model

## Abstract

It remains difficult for mobile robots to continue accurate self-localization when they are suddenly teleported to a location that is different from their beliefs during navigation. Incorporating insights from neuroscience into developing a spatial cognition model for mobile robots may make it possible to acquire the ability to respond appropriately to changing situations, similar to living organisms. Recent neuroscience research has shown that during teleportation in rat navigation, neural populations of place cells in the cornu ammonis-3 region of the hippocampus, which are sparse representations of each other, switch discretely. In this study, we construct a spatial cognition model using brain reference architecture-driven development, a method for developing brain-inspired software that is functionally and structurally consistent with the brain. The spatial cognition model was realized by integrating the recurrent state—space model, a world model, with Monte Carlo localization to infer allocentric self-positions within the framework of neuro-symbol emergence in the robotics toolkit. The spatial cognition model, which models the cornu ammonis-1 and -3 regions with each latent variable, demonstrated improved self-localization performance of mobile robots during teleportation in a simulation environment. Moreover, it was confirmed that sparse neural activity could be obtained for the latent variables corresponding to cornu ammonis-3. These results suggest that spatial cognition models incorporating neuroscience insights can contribute to improving the self-localization technology for mobile robots. The project website is https://nakashimatakeshi.github.io/HF-IGL/.

## 1 Introduction

The intersection of neuroscience and robotics has been re-emphasized as an approach to advancing artificial intelligence in recent years (Zador et al., 2023). The complex, uncertain, and dynamic environments in which actual robots operate require the artificial intelligence embedded in robots to acquire adaptability, even if it involves significant dynamic changes that have never been experienced before. From the neuroscience perspective, the hippocampal formation is involved in the cognitive map that enables flexible behavior in response to environmental changes, a concept named by Tolman (1948). Indeed, it has been revealed that the cornu ammonis-3 (CA3) subregion plays a crucial role when the hippocampus of rats perceive **teleportation**, where the environment surrounding oneself changes dynamically and suddenly (Jezek et al., 2011). Therefore, this study incorporates these findings to propose a spatial cognition system for mobile robots capable of dealing with teleportation, also known as the **kidnapped robot problem**.

Models for self-localization referencing the hippocampus have been proposed but could not consider the hippocampus's subregions. RatSLAM is a probabilistic method inspired by the function of integrating path integration and egocentric local view in the rat's hippocampus (Milford et al., 2004). However, this method did not consider the structure of the hippocampal subregions. The reason for this lack of consideration is that the roles and information processing, i.e., functions of the hippocampus's subregions have not been fully elucidated even today, and the only consensus lies in its structure, making it challenging to model them explicitly. However, with the recent development of deep neural networks, learning functions and representations from data by mimicking the structure is possible.

It remains difficult for mobile robots to continue accurate self-localization when they are suddenly teleported to a location that is different from their beliefs during navigation. The situation where a robot undergoes teleportation is called the kidnapped robot problem. The difficulty of the self-localization problem is classified according to a combination of the robot's believed self-position and its true self-position at the start (Zhang et al., 2009). Self-localization from a state where the belief and true positions are the same is called pose tracking or position tracking and is the simplest task. Self-localization from a state in which there is no information about the robot's position and the robot's belief is uniform across the environment is called global self-localization. When a robot believes it is in a specific place, but it is not, the problem is called the kidnapped robot problem. This scenario, known as the kidnapped robot problem, is the most challenging scenario for self-localization. As the name suggests, the kidnapped robot problem assumes that a robot navigating an environment is suddenly teleported to a different location.

Conventional methods involve two steps to tackle the kidnapped robot problem: detecting the kidnapping and proposing a hypothesis that narrows the search area. Kidnapping detection uses the entropy of the self-location distribution uncertainty. Then, the search area can be narrowed using a relatively robust state estimation method such as the finite impulse response filter (Pak et al., 2015; Pak and Ahn, 2023) or a multivariate Gaussian distribution centered on the estimated position when kidnapping is detected (Meng et al., 2020). Another method uses sparse topological maps composed of highly distinctive global features (Choi et al., 2012). The strategy of adding functions appropriate to the degree of variation can rapidly increase engineering costs when adapting to changing environments. Hence, investigating methods based on hippocampal formation has become essential to adapting to changing situations.

Self-localization is an essential capability of robots for navigating an environment. Thus, it has been a subject of engineering research in the field of robotics. Localization is the task of estimating one's constantly changing position from the sequence information of perceptions by the robot's sensors and a map of the environment. A probabilistic approach is developed to address the uncertainty of the environment and the perception sensors (Thrun, 2002). The probabilistic approach was formulated using a state-space model that allows sequential computation and is used in robots that act adaptively over long periods. The probability distribution of the state is inferred through alternating updates based on behavior and observation, which is referred to as a Bayes filter. The method of approximating a probability distribution with a set of particles is called Monte Carlo localization (MCL) (see Section 2.1). It is preferred for its capacity to represent complex distribution shapes and its simplicity in calculating posterior probabilities (Murphy and Russell, 2001). Ueda et al. (2004) proposed a Monte Carlo Localization using expansion resetting (EMCL), which expands the particle set when the likelihood of sensor values decreases, demonstrating that it enables more robust self-localization. However, the update function for the state distribution had to be designed by the engineer beforehand.

Methods have been proposed that utilize deep neural networks to directly learn the update functions based on behavior and observation in state-space models from sequence data, among which models capable of predicting the dynamics of the external world are called world models. The recurrent state-space model (RSSM), a sequential variational autoencoder (VAE) for state representation learning, acquires robust representations from sequences of raw images by incorporating both deterministic and stochastic transition components into the dynamics model (Hafner et al., 2019b) (see Section 2.2). Furthermore, the Multimodal RSSM (MRSSM), which enables the learning of state representations from multimodal information by adopting a Multimodal VAE, has been proposed (Komura et al., 2023). These techniques enable the learning of dynamics from data by pre-defining the network's structure.

In addition, efforts have been made to consolidate the extensive field of neuroscience with reference architectures to model the brain structure. Yamakawa (2021) introduced brain reference architecture (BRA)-driven development (see Section 2.3), a software development approach that enables the construction of software architectures inspired by the structure and function of the brain. Similarly, Taniguchi T. et al. (2022) and Taniguchi et al. (2020) proposed a method called the whole brain probabilistic generative model (WB-PGM), which aims to realize a cognitive architecture by integrating modules constructed with probabilistic generative models through the neuro-symbol emergence in the robotics toolkit (Neuro-SERKET). These techniques enable the construction of brain-inspired computational models, which is sought after in the field of machine learning.

Based on the above, the problem statement, hypotheses, and research questions of this study are as follows:

### 1.1 Problem statement

Neuroscience insights are necessary to address the issue that current machine learning systems are designed solely to realize functions. The kidnapped robot problem requires mobile robots to have a wide range of adaptability. Conventional engineering approaches to this problem involve adding functions during the design phase. In the case of mobile robots, this involves adding effective self-localization methods against the kidnapped robot problem and switching methods upon detecting kidnapping. However, this design philosophy cannot handle situations that designers do not anticipate. This study aimed to achieve adaptability within a single spatial cognition model by referencing the brains of biological organisms.

### 1.2 Hypothesis

This study hypothesized that the brain's structure is closely related to its function. In particular, this study was based on the hypothesis that the distinctive structure of the hippocampus (see Section 2.4) supports spatial cognition. Therefore, we tested whether a self-localization model emulating the structure of the hippocampus could improve the self-localization performance in the kidnapped robot problem.

### 1.3 Research question

The research question of this study aimed to elucidate the mechanisms of spatial cognition in organisms by constructing computational models that mimic the structure of the hippocampus. How do organisms adapt to changes in situations that they have never experienced based on their learning?

### 1.4 Approach

In this study, we construct a global self-localization model that is consistent with the structure and function of the hippocampus, using the BRA-driven development methodology. The proposed method integrates two modules, MCL and RSSM, within the Neuro-SERKET framework. The medial entorhinal cortex (MEC), which processes allocentric information, is modeled with the MCL module to infer positions in the map coordinate system. In contrast, the hippocampus, which integrates two different types of information state representations, and the lateral entorhinal cortex (LEC), which processes egocentric information, are modeled with the RSSM module that learns state representations from first-person images. We evaluated and compared the MCL, EMCL and proposed methods that are integrated in different ways for global self-localization and the kidnapped robot problem.

The main contributions of this study are as follows:

1. We realize a self-localization estimation model that is functionally and structurally consistent with the hippocampal formation using the methodology of BRA-driven development.
2. We showed that the self-localization performance in the kidnapped robot problem is improved through a simulation experiment.

The remainder of this article is organized as follows: Section 2 describes the self-localization method and RSSM that are the basis of this research and describes the BRA-driven development, which is the development methodology for brain reference software, and the neuroscientific knowledge of hippocampal formation. Section 3 describes the architecture of the proposed model and self-localization method. Section 4 describes the experimental setup and the results. Section 5 discusses the relationships in the hippocampus. Finally, Section 6 provides a conclusion.

## 2 Background

In this section, we describe MCL, a conventional self-localization method, and RSSM, a type of world model. Both are employed as modules in the hippocampus-referencing global self-localization model proposed in this study. We then discuss the BRA-driven development methodology that guided the design of our model. Finally, we elaborate on the neuroscientific knowledge of hippocampal formation that our model references.

### 2.1 Self-localization in robots

**Figure 1:**
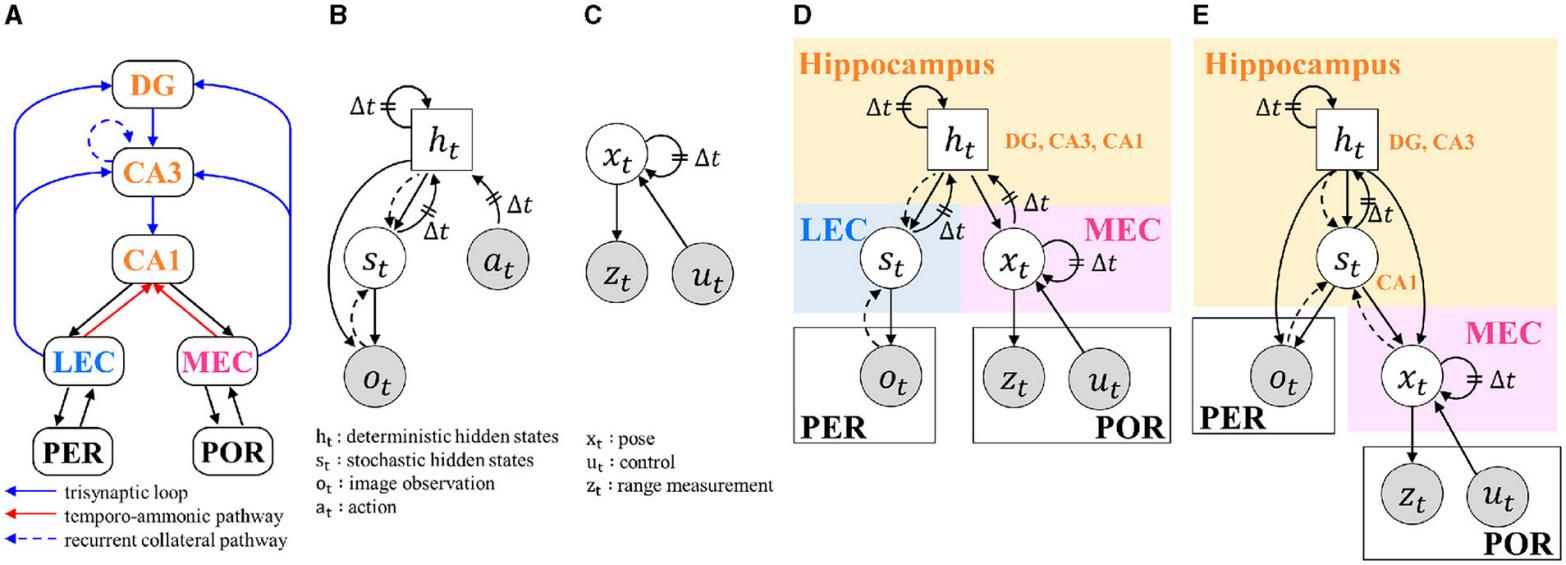
BIF and graphical models: **(A)** ROI of this study and its BIF. DG, dentate gyrus; CA3, and CA1, cornu ammonis-3 and -1; LEC and MEC, lateral and medial entorhinal cortex; PER, perirhinal cortex; POR, postrhinal cortex; **(B)** RSSM without rewards; **(C)** MCL; **(D)** Model 1, which models the hippocampal DG, CA3, and CA1 with a single random variable *h*_*t*_; **(E)** Model 2, which models the hippocampal DG and CA3 with the random variable *h*_*t*_ and CA1 with *s*_*t*_. In graphical models, gray circles indicate observable variables. Circles represent stochastic variables, and squares represent deterministic variables. Solid arrows represent the generative process, and dashed arrows represent the inference process. Δ*t* indicates dependence on the next time step.

The problem of a robot estimating its position using its sensory information is called self-localization. When an environmental map is not provided, the problem is called simultaneous localization and mapping (SLAM) (Thrun, 2002). Robots often have two types of sensors: external sensors, such as cameras and laser scanners, to observe the external environment, and internal sensors, such as odometry and inertial measurement units, to perceive and control their movements. This problem has two approaches: an optimization approach and a Bayes filter approach. The optimization approach calculates the entire trajectory by optimizing the pose graph of the self-pose constraints constructed from observation and motion information. Note that the term pose includes information on both position and orientation. The Bayes filter approach is a state-space model that sequentially calculates the states (self-position distribution) by assuming a Markov property. The latter is a type of probabilistic generative model (PGM) that represents the dependence of random variables as a graphical model (Figure 1C). In the following section, we describe the Bayes filter approach in detail.

Self-localization using the Bayes filter approach is the problem of estimating the conditional probability (belief) of the self-pose given perceptual sequence information, as shown in the following Equation (1):


(1) $$\begin{array}{l} \text{Bel}(t)=p(x_{1:t}|z_{1:t},u_{1:t})\\ \;\propto \underset{\text{likelihood}}{\underset{︸}{p(z_{t}|x_{1:t},z_{1:t-1},u_{1:t})}}\underset{\text{prior}}{\underset{︸}{p(x_{1:t}|z_{1:t-1},u_{1:t})}}\;(\text{Bayes}\prime \text{ theorem})\\ \;=p(z_{t}|x_{1:t},z_{1:t-1},u_{1:t})p(x_{t}|x_{1:t-1},z_{1:t-1},u_{1:t})p(x_{1:t-1}|z_{1:t-1},u_{1:t})\\ \;=p(z_{t}|x_{t})p(x_{t}|x_{t-1},u_{t})p(x_{1:t-1}|z_{1:t-1},u_{1:t-1})\;(\text{Markov property})\\ \;=\underset{\text{measurement update}}{\underset{︸}{p(z_{t}|x_{t})}}\underset{\text{prediction}}{\underset{︸}{p(x_{t}|x_{t-1},u_{t})}}\text{Bel}(t-1) \end{array}$$

Here, *x*_1:*t*_ is the trajectory of the self-pose, *z*_1:*t* − 1_ and *u*_1:*t*_ are the measurement values of the external and internal sensors, respectively. Belief is updated by predicting the prior through control update and calculating the posterior through measurement update (i.e., Bayes filter). Since the state *x* assumes the Markov property, and state *x*_*t*_ is not influenced by the state before *x*_*t*−1_, belief updates are calculated sequentially.

The map format used in this study was a two-dimensional occupancy grid map. An occupancy grid map discretizes the space into a grid, and each grid cell represents the locations occupied by obstacles and unoccupied free locations as binary information (**Figure 6**). When a pose *x* on the occupied grid map is given, the likelihood of measurement *p*(*z*_*t*_|*x*_*t*_) can be calculated from the relative relationship between the occupied cell and its pose. Note that the notation for map *m* is often explicitly stated as *p*(*z*_*t*_|*x*_*t*_, *m*), but it is omitted for brevity.

MCL is a method for approximately estimating the probability distribution of self-pose using a particle set sampled using the Monte Carlo method. The implementation becomes simpler, even when the observation model or transition model is nonlinear by representing the distribution of particles. However, the performance of self-localization depends on the number of particles, and the larger the environment, the more particles are required; however, this becomes a trade-off with computational resources.

Self-localization technology is used in autonomous driving and service robotics and is necessary for developing agents that act in the real world.

### 2.2 Recurrent state-space model: one of the world models

A world model refers to a model of the external world that an agent maintains internally and is used for perceptual prediction and behavior generation. Early mobile-robot research sometimes referred to environmental maps as occupancy grid maps (Thrun, 2002). In recent years, deep learning has been used to predict future perceptions by learning representations and their dynamics in the state-space (Ha and Schmidhuber, 2018).

The RSSM, a world model, can learn policies with high sample efficiency compared to end-to-end methods (Hafner et al., 2019b). This efficiency is accomplished by leveraging episodes imagined by the learned world model for reinforcement learning (Hafner et al., 2019a, 2020, 2023; Wu et al., 2023). RSSM is a type of probabilistic generative model that illustrates the dependence of random variables as a graphical model in Figure 1B. A stable state can be maintained over multiple steps from a noisy, high-dimensional observed image *o*_*t*_ using both deterministic variable *h*_*t*_ and stochastic variable *s*_*t*_ as latent variables. Circles indicate probabilistic variables, squares indicate deterministic variables, solid lines indicate generative models and dotted lines indicate inference models. The RSSM generation and inference models are expressed as following Equation (2):


(2) $$\begin{array}{ll} \text{ Generative model:} & h_{t}=f^{GRU}(h_{t-1},s_{t-1},a_{t-1})\\ \; & s_{t}\sim p(s_{t}|h_{t})\\ \; & o_{t}\sim p(o_{t}|h_{t},s_{t})\\ \text{ Inference model:} & s_{t}\sim q(s_{t}|o_{t},h_{t}) \end{array}.$$

RSSM is a deep generative model approximating probabilistic dependencies between latent variables using a deep neural network. In machine learning, the top-down prediction generation and bottom-up model updating are called inferences. In the RSSM, the state *h*_*t*_ is predicted from the states and action at the previous time step by a gated recurrent unit (GRU) responsible for the deterministic dynamics in state-space, followed by the prediction of the probabilistic distribution of *s*_*t*_. Subsequently, the inference model, which is trained within the framework of VAE, infers the latent variables from observations. The parameters of each model were optimized by backpropagation to maximize the log marginal likelihood, as shown in the following Equation (3):


(3) $$\begin{array}{c} \ln p(o_{1:T}|a_{1:T})\\ \geq \sum_{t=1}^{T}(\underset{\text{reconstruction}}{\underset{︸}{{\text{E}}_{q(s_{t}|o_{\leq t},a_{<t})}[\ln p(o_{t}|s_{t})]}}\\ -\underset{\text{complexity}}{\underset{︸}{{\text{E}}_{q(s_{t}|o_{\leq t},a_{<t})}[\text{KL}[q(s_{t}|o_{\leq t},a_{<t})\|p(s_{t}|s_{t-1},a_{t-1}]}}) \end{array}$$

The evidence lower bound (ELBO) is the right side derived using Jensen's inequality. The first term is called reconstruction, in which the prediction error of observation *o*_*t*_ is minimized. The second term is called complexity because it penalizes the complexity of the model that minimizes the prediction error of states *s*_*t*_ in the state space.

### 2.3 Brain reference architecture-driven development

BRA-driven development is a methodology for building software based on brain architecture (Yamakawa, 2021). It accepts that neuroscience knowledge is still insufficient to elucidate the whole picture and hypothetically construct a software architecture using anatomical structures as constraints. A structure-constrained interface decomposition (SCID) method was used to design software that was consistent with the structure and function of the brain obtained through neuroscience. The software consisted of the following three steps.

Step 1: BIF construction
Step 2: Consistent determination of ROI and TLF
Step 3: HCD creation
  Step 3-A: Enumeration of candidate HDCs
  Step 3-B: Rejection of HCDs that are inconsistent with scientific knowledge

In Step 1, brain information flow (BIF), a graph representing the anatomical structure of neural circuits in the brain at the mesoscopic level, is constructed based on research in mammalian neuroscience. In Step 2, a specific region of interest (ROI) in the brain and the top-level function (TLF) to be performed by the brain region are determined according to the purpose of each system. In Step 3, hypothetical component diagrams (HCD) that are decomposed into functional units to satisfy the TLF are constructed under the structural constraints of the BIF graph. This hypothetical component diagram is repeatedly enumerated and rejected based on knowledge from various fields, such as neuroscience, cognitive psychology, evolutionary theory, and developmental theory.

WB-PGM (Taniguchi T. et al., 2022) proposed a modular approach to implementing HCD using a probabilistic generative model. We can integrate the modules of the probabilistic generative model and enable each module to continue inference continuously using neuro-symbol emergence in the robotics toolkit (Neuro-SERKET) (Taniguchi et al., 2020). This framework enables the integration of different modules, including those that use different inference methods for approximate posterior distributions, such as sampling and variational inference. Generation-Inference Process Allocation (GIPA) is a method for realizing HCD using a probabilistic generative model. In GIPA, the components and relationships are assigned to random variables, generation processes, and inference processes. Based on these methodologies, Taniguchi A. et al. (2022) proposed the hippocampal formation-inspired probabilistic generative models. In this case, loop circuits exist in some of the neural circuits of the brain; however, in the probabilistic graphical model, they need to be directed-acyclic graphs. Assuming that there is a time delay in signal transmission, we express the loop circuit in the brain by using Δ*t* to represent the transition of the generation process to the next time step. It should be noted that the graphical model of the integrated model does not have the strictly same structure as BIF, and supplementing paths and combining multiple brain regions into a single latent variable is done hypothetically during modeling. In the case where the direction of the generative model's arrow and the projection of the BIF are in opposite directions, it can be interpreted that during learning, the error propagates in the opposite direction of the arrow of the generative model.

### 2.4 Neuroscientific knowledge of hippocampal formation

In the field of neuroscience, there has been an accumulation of knowledge on how the hippocampal formation of organisms contributes to spatial cognition. Tolman (1948) demonstrated that rats searching for food within an environment learn spatial representations that enable flexible behavior in reaction to blockages of familiar route, rather than in response to simple stimulus-response associations, and named this spatial representation a cognitive map. Later, it was reported that various cells that support the cognitive map, such as place cells, exist in the hippocampal formation (O'Keefe and Nadel, 1979).

The hippocampal formation is part of the limbic system and involves episodic memory formation and spatial cognition. The ROI in this study is shown in Figure 1A. It consists of the hippocampus, which includes cornu ammonis-1, -3, and dentate gyrus (CA1, CA3, DG), the LEC and MEC, perirhinal cortex (PER) and postrhinal cortex (POR). There are two main neural circuits within this region (Knierim, 2015). One is the trisynaptic loop, which projects from the entorhinal cortices (EC) to the DG, CA3, and CA1 through a perforant path, a mossy fiber pathway, and a Schaffer collateral pathway. The other is a direct pathway that projects from the EC to CA1, called the temporo-ammonic pathway. In addition to these neural circuits, CA3 is known to have a recurrent collateral pathway that projects to its region. The main output from the hippocampus is the projection from CA1 to the MEC and LEC.

Several studies suggest that the subregions of the hippocampus, particularly CA1, and CA3, possess distinct characteristics and may serve different functions (Farovik et al., 2010). In CA1, events are associated with a mechanism in which part of the neural ensemble is shared (Cai et al., 2016). On the other hand, the representation in CA3 is sparser (Leutgeb et al., 2004) and more coherent (Lee et al., 2004) compared to that of CA1, and it supports a distinguishing function (Kesner and Rolls, 2015). According to Dimsdale-Zucker et al. (2018), CA1 connects objects in different contexts, whereas CA2, CA3, and DG may play a role in distinguishing objects in the same context. Significantly, recent research has shown that, during the teleportation of rats, sparse representations in the CA3 region of the hippocampus discretely switch, flickering within the theta cycle (Jezek et al., 2011).

Inputs to the hippocampus are made through the LEC and MEC. The LEC and MEC are thought to handle different information, but the specifics of their classifications may vary. In early studies, MEC was expressed as spatial information and LEC as non-spatial information (Hargreaves et al., 2005); however, later, MEC processed its own operational (path integration) information, and LEC processed external observation information (Deshmukh and Knierim, 2011). Wang et al. (2020) claimed MEC processes allocentric information, while LEC processes egocentric information.

Computational theories and models of hippocampal formation have explained its ability to integrate and relate disparate information. In cognitive map theory (CMT) (O'Keefe and Nadel, 1979), event (item) is associated with spatial and temporal position, and in the binding of items and contexts (BIC) theory (Eichenbaum et al., 2007), event(item) is tied to the context.

## 3 Proposed method

In this study, we constructed global self-localization models by integrating the following two models. The first is the RSSM, which extracts features from images and constructs them as representations of time-series dynamics. The second method is MCL, which is an engineering-based self-localization method based on the occupancy grid maps. They were integrated into the framework of Neuro-SERKET because RSSM and MCL are both probabilistic generative models. The RSSM is responsible for processing egocentric visual representations (i.e., LEC) and integrating egocentric and allocentric information (i.e., CA1, CA3, DG). MCL is responsible for processing allocentric spatial representations (i.e., MEC). There is a debate on how allocentric representations are represented in the brain (Ekstrom et al., 2014). However, this study assumed that allocentric representations were robot poses within an occupied grid map.

The method of integrating RSSM and MCL was determined based on the structure of the hippocampal formation. As discussed in the SCID method, multiple structures are considerable (Section 2.3). Thus, two models with varying degrees of granularity were constructed, and integrated graphical models are shown in Figures 1D, E. Here, the variables *o*_*t*_, *z*_*t*_, and *u*_*t*_, which are encircled in gray, are observed variables that represent an image from the RBG camera, range measurement from the LIDER sensor, and control values, respectively. The solid and dotted lines in the graphical model represent the generative and inferential models, respectively. Model 1 models the hippocampal DG, CA3, and CA1 using one random variable, *h*_*t*_. Model 2 models hippocampal DG and CA3 with random variables *h*_*t*_ and CA1 with *s*_*t*_.

From the perspective of a state-space model, Model 1 associates *x*_*t*_ as the input and *o*_*t*_ as the output via the state-space. Treating signals related to control as inputs is a straightforward approach to conventional state-space models. In contrast, in Model 2, *x*_*t*_ and *o*_*t*_ both correspond to outputs (observations) and use Multimodal RSSM (MRSSM) (Komura et al., 2023) to obtain the latent representation shared by the two modalities. Here, the latent variable *s*_*t*_ corresponds to the CA1 region, and the mechanism for linking events is similar to each other.

The generative and inference models are expressed as Models 1 and 2, respectively, as shown in Equation (4).


(4) $$\begin{array}{lll} \; & \text{Model 1} & \text{Model 2}\\ \text{ Generative model: } & h_{t}=f^{GRU}\left(h_{t-1},s_{t-1},x_{t-1}\right) & h_{t}=f^{GRU}\left(h_{t-1},s_{t-1}\right)\\ \; & s_{t}\sim p\left(s_{t}|h_{t}\right) & s_{t}\sim p\left(s_{t}|h_{t}\right)\\ \; & o_{t}\sim p\left(o_{t}|s_{t}\right) & o_{t}\sim p\left(o_{t}|h_{t},s_{t}\right)\\ \; & x_{t}\sim p\left(x_{t}|h_{t}\right) & x_{t}\sim p\left(x_{t}|h_{t},s_{t}\right)\\ \; & z_{t}\sim p\left(z_{t}|x_{t}\right) & z_{t}\sim p\left(z_{t}|x_{t}\right)\\ \; & x_{t}\sim p\left(x_{t}|x_{t-1},u_{t}\right) & x_{t}\sim p\left(x_{t}|x_{t-1},u_{t}\right)\\ \text{ Inference model: } & s_{t}\sim q(s_{t}|o_{t},h_{t}) & s_{t}\sim q(s_{t}|o_{t},x_{t},h_{t}) \end{array}$$

Model 1 treats *x*_*t*_ as input information for the state-space model. Since conventional state-space models do not include a model that predicts input *x*_*t*_ from the state, the generative model *p*(*x*_*t*_|*h*_*t*_) is added to Model 1. Since a direct connection from the hippocampus to the PER is not assumed, *h*_*t*_ cannot be given to the generative model *p*(*o*_*t*_|*s*_*t*_) in Model 1. The MCL observation model *p*(*z*_*t*_|*x*_*t*_) and transition model *p*(*x*_*t*_|*x*_*t*−1_, *u*_*t*_) were modeled in advance using an inductive bias; hence, training was not necessary. However, the generation and inference models of the RSSM and MRSSM modules are approximated by neural networks, and it is necessary to train the neural network parameters from the training data. Therefore, the RSSM and MRSSM modules are trained independently. In the next section, we describe the RSSM and MRSSM modules.

### 3.1 Architecture

**Figure 2:**
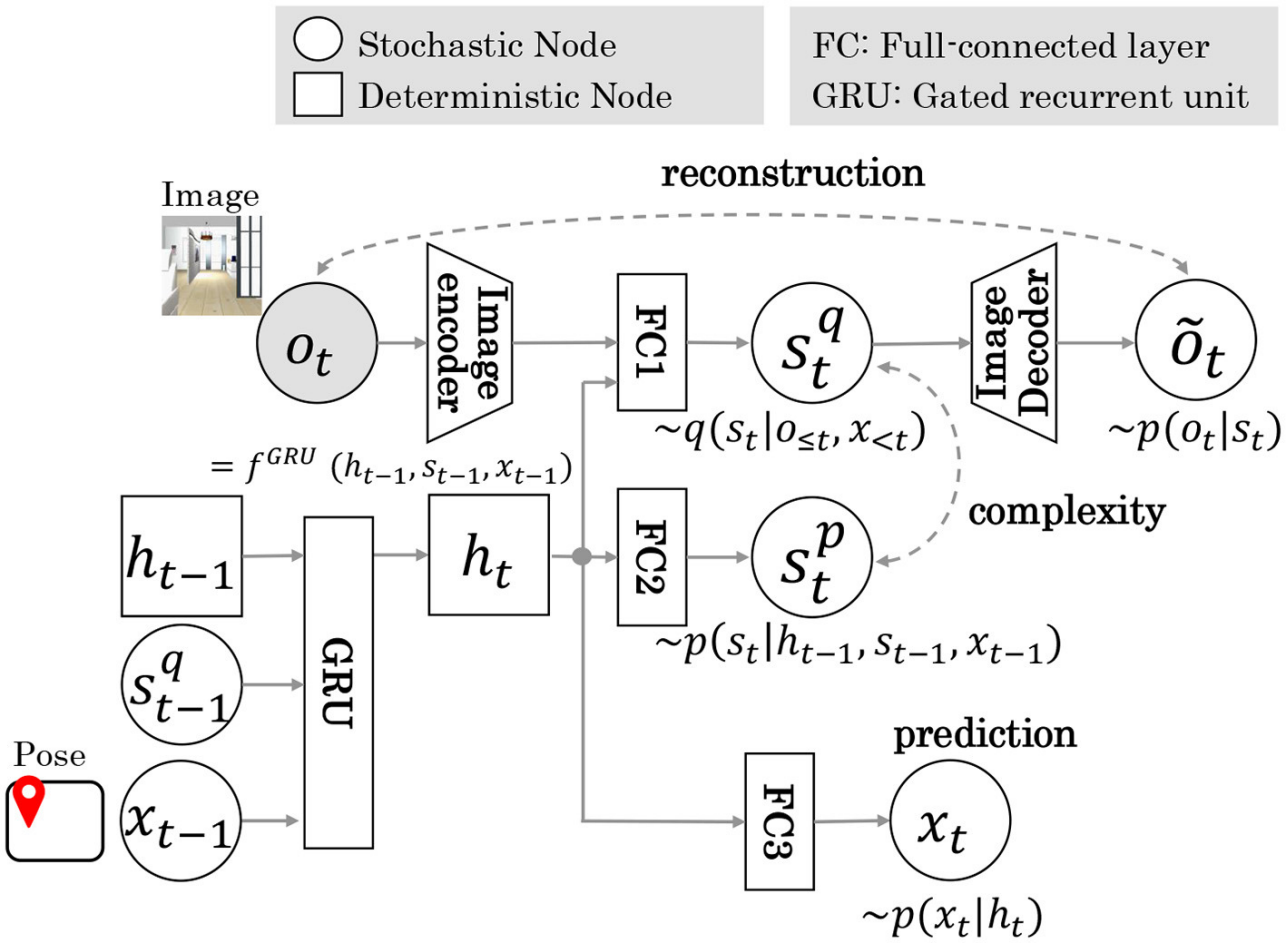
The RSSM architecture of Model 1. Dotted arrows indicate loss during training. *x*_*t*−1_ is input to GRU unit. The terms reconstruction, complexity, and prediction indicate training loss function.

Figure 2 shows the RSSM architecture diagram of Model 1. In Model 1, pose *x* inferred by MCL is input to GRU, which is responsible for the state transition model of RSSM. Here $\tilde{o_{t}}$ is the reconstructed image information. $s_{t}^{p}$ is a prior distribution sampled from probability distribution *p*(*s*_*t*_|*h*_*t*_). $s_{t}^{q}$ is the posterior distribution sampled from the probability distribution *q*(*s*_*t*_|*h*_*t*_, *o*_*t*_). The parameters of these models were trained in a self-supervised learning framework based on the sequence data of the image and pose estimated by MCL. The objective function is the maximization of the log marginal likelihood of the joint distribution ln*p*(*o*_1:*T*_, *x*_1:*T*_). Here, *T* denotes the length of the training data sequence. This formula, divided into reconstruction, complexity, and prediction terms using Jensen's inequality, is shown in Equation (5).


(5) $$\begin{array}{l} \text{Model 1:}\\ \;\ln p(o_{1:T},x_{1:T})≜\ln{\text{E}}_{q(h_{1:T},s_{1:T}|x_{1:T})}\left[\prod_{t=1}^{T}p(o_{t}|s_{t})p(x_{t}|h_{t})\right]\\ \;\geq \sum_{t=1}^{T}(\underset{\text{reconstruction}}{\underset{︸}{{\text{E}}_{q(h_{t},s_{t}|o_{\leq t},x_{<t})}\left[\ln p(o_{t}|s_{t})\right]}}+\underset{\text{prediction}}{\underset{︸}{{\text{E}}_{q(h_{t},s_{t}|o_{\leq t},x_{<t})}\left[\ln p(x_{t}|h_{t})\right]}}\\ \;\\ \;\underset{\text{complexity}}{\underset{︸}{-\begin{array}{l} \text{E}\left[\text{KL}\left[q(s_{t}|o_{\leq t},x_{<t})||p(s_{t}|h_{t-1},s_{t-1},x_{t-1})\right]\right])\\ q(h_{t-1},s_{t-1}|o_{\leq t-1},x_{<t-1}) \end{array}}} \end{array}$$

**Figure 3:**
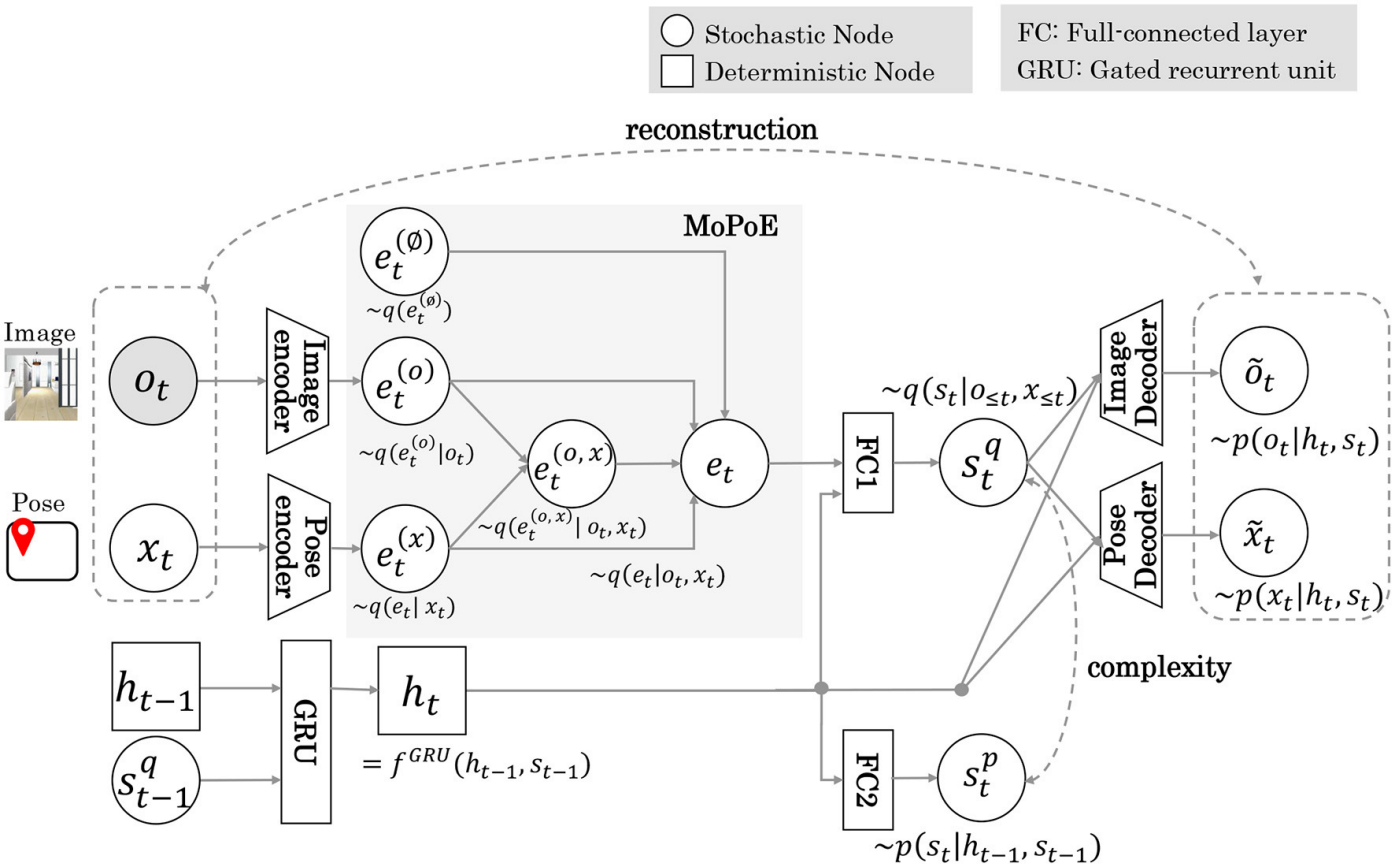
The MRSSM architecture of Model 2. Gray areas indicate the integration of multimodal observation by the MoPoE scheme.

Figure 3 shows the MRSSM architecture of Model 2. Details of the architecture are provided in Table S1. In Model 2, pose *x* inferred by MCL is input to the encoder as a multimodal observation of the images and poses. The latent representation shared by the multimodal observations was approximated by integrating each expert using MoPoE (Komura et al., 2023). The *e*_*t*_ embedding vector is calculated by the following Equation (6).


(6) $$\begin{array}{l} e_{t}\sim \frac{1}{2^{M}}\underset{{𝕆}_{t}\in P\left({𝕆}_{t}\right)}{\sum }q\left(e_{t}|{𝕆}_{t}\right) \end{array}$$

Here, 𝕆_*t*_ is a subset of {∅, *o*_*t*_, *x*_*t*_} and *M* = 2 is the number of modalities. A latent variable integrating both modalities allows cross-modal inference from *o*_*t*_ to *x*_*t*_ and vice versa. Similarly to Model 1, the objective function, which maximizes the log marginal likelihood of the joint distribution, is represented by the following Equation (7).


(7) $$\begin{array}{l} \text{Model 2:}\\ \;\ln p(o_{1:T},x_{1:T})≜\ln{\text{E}}_{q(h_{1:T},s_{1:T})}\left[\prod_{t=1}^{T}p(o_{t}|h_{t},s_{t})p(x_{t}|h_{t},s_{t})\right]\\ \;\geq \sum_{t=1}^{T}(\underset{\text{reconstruction}}{\underset{︸}{{\text{E}}_{q(h_{t},s_{t}|o_{\leq t},x_{\leq t})}\left[\ln p(o_{t}|h_{t},s_{t})\right]+{\text{E}}_{q(h_{t},s_{t}|o_{\leq t},x_{\leq t})}\left[\ln p(x_{t}|h_{t},s_{t})\right]}}\\ \;\underset{\text{complexity}}{\underset{︸}{\begin{array}{l} -\text{E}\left[\text{KL}\left[q(s_{t}|o_{\leq t},x_{\leq t})||p(s_{t}|h_{t-1},s_{t-1})\right]\right])\\ \;q(h_{t-1},s_{t-1}|o_{\leq t-1},x_{\leq t-1}) \end{array}}} \end{array}$$

### 3.2 Self position estimation

In this section, we describe the method for sequentially inferring self-pose *x*_*t*_ in Models 1 and 2. The distribution of all the latent variables Bel(*t*) = *p*(*x*_1:*t*_, *h*_1:*t*_, *s*_1:*t*_|*o*_1:*t*_, *z*_1:*t*_, *u*_1:*t*_) is called a belief. It can be represented by the sequential update formulas shown in Equations (8, 9), using Bayes' theorem and the Markov property.


(8) $$\begin{array}{ll} \text{Model 1: } & \text{Bel}\left(t\right)\approx \underset{\text{MCL}}{\underset{︸}{p\left(z_{t}|x_{t}\right)p\left(x_{t}|x_{t-1},u_{t}\right)}}\underset{\text{RSSM}}{\underset{︸}{p\left(x_{t}|h_{t}\right)q\left(s_{t}|o_{t},h_{t}\right)f^{GRU}\left(h_{t-1},s_{t-1},x_{t-1}\right)}}\text{Bel}\left(t-1\right) \end{array}$$

(9) $$\begin{array}{ll} \text{Model 2: } & \text{Bel}\left(t\right)\approx \underset{\text{MCL}}{\underset{︸}{p\left(z_{t}|x_{t}\right)p\left(x_{t}|x_{t-1},u_{t}\right)}}\underset{\text{MRSSM}}{\underset{︸}{p\left(x_{t}|s_{t},h_{t}\right)q\left(s_{t}|o_{t},h_{t}\right)f^{GRU}\left(h_{t-1},s_{t-1}\right)}}\text{Bel}\left(t-1\right) \end{array}$$

**Figure 4:**
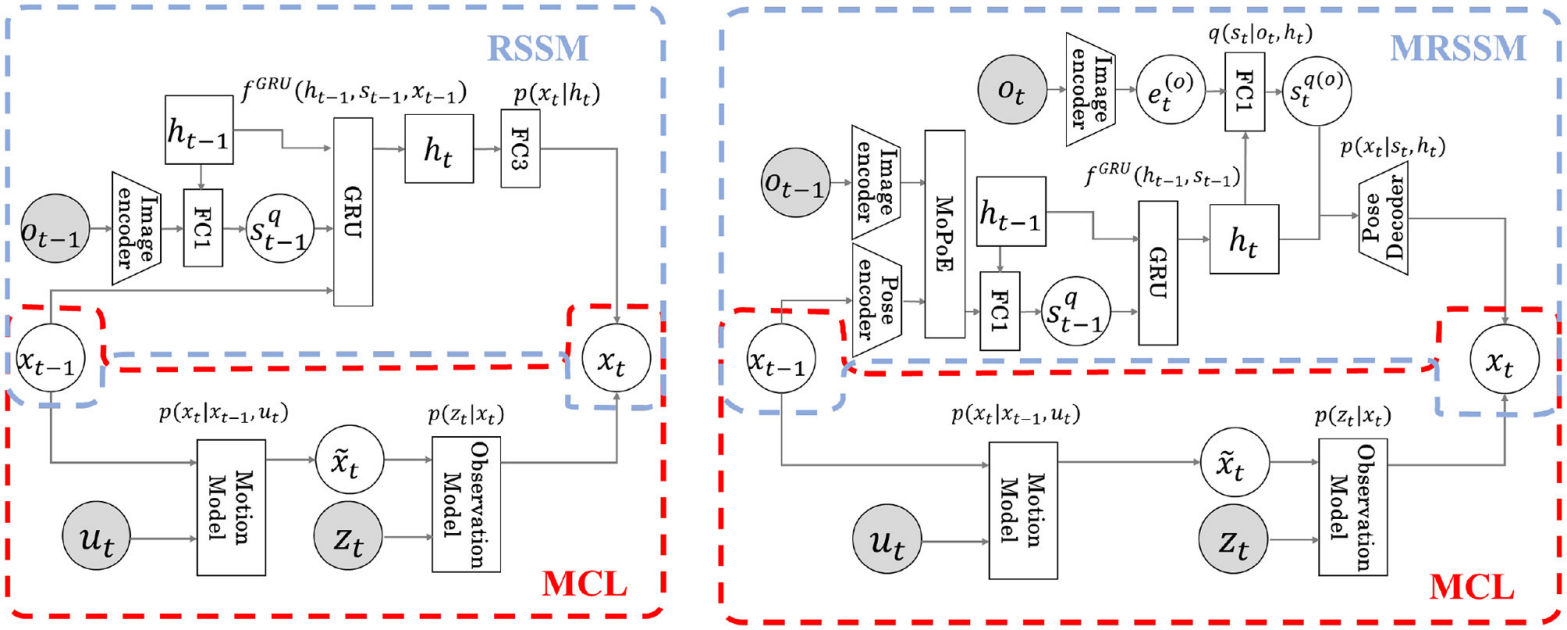
Flow diagram of estimation of next time pose*x*_*t*_ for each model. Both models integrate the RSSM (blue dotted line) and the MCL (red dotted line) by sharing the latent variable *x* in the Neuro-SERKET framework. In Model 1, previous time pose *x*_*t*−1_ is input to the GRU modeling the transition, and the latent variable *h*_*t*_ at the next time is predicted. Then, *x*_*t*_ is predicted from *h*_*t*_ **(Left)**. In Model 2, using an inference model, *x*_*t*−1_ is used to infer the latent variable *s*_*t*−1_. The transition model then predicts the latent variable *h*_*t*_ for the next time step, and *x*_*t*_ is predicted by cross-modal inference using *o*_*t*_ via latent variables *s*_*t*_ and *h*_*t*_ **(Right)**.

The detailed calculation formulas are shown in Equations (S2) and (S3), respectively. The computation flow diagrams for pose *x*_*t*_ in Models 1 and 2 are shown in Figure 4. The area surrounded by the red dotted line is the MCL module, and the blue dotted line is the RSSM or MRSSM. Through integration using Neuro-SERKET, the probabilistic variable pose *x* is shared with MCL and RSSM or MRSSM modules. The pose *x* is inferred in each module and eventually integrated. The MCL operation is the same for both Models 1 and 2. The operation of MCL involves prediction through the motion model followed by observation update through the observation model. However, RSSM and MRSSM differ for Models 1 and 2. In RSSM for Model 1, the pose at the previous time step *x*_*t*−1_ is input into the GRU, progressively predicting the latent variable of *h*_*t*_ and *s*_*t*_. Contrarily, in MRSSM for Model 2, *x*_*t*−1_ is used to infer *s*_*t*−1_ serving as input to the GRU. Subsequently, utilizing the predicted *h*_*t*_ by the GRU, cross-modal inference is employed to infer the pose through the stochastic latent variable $s_{t}^{q\left(o\right)}$, which is inferred from the observed image *o*_*t*_ without using the pose *x*_*t*_. The stochastic latent variable at time *t* is recalculated in the next time step using both modal information from the image and the pose. Finally, in both Model 1 and 2, the integrated distribution of the pose at time t *x*_*t*_ is represented by a particle set sampled at 75% from the MCL and the remaining 25% from RSSM or MRSSM.

## 4 Experiment

Experiments were conducted in a simulation environment to evaluate the global self-localization performance, including the kidnapped robot problem.

### 4.1 Conditions

**Figure 5:**
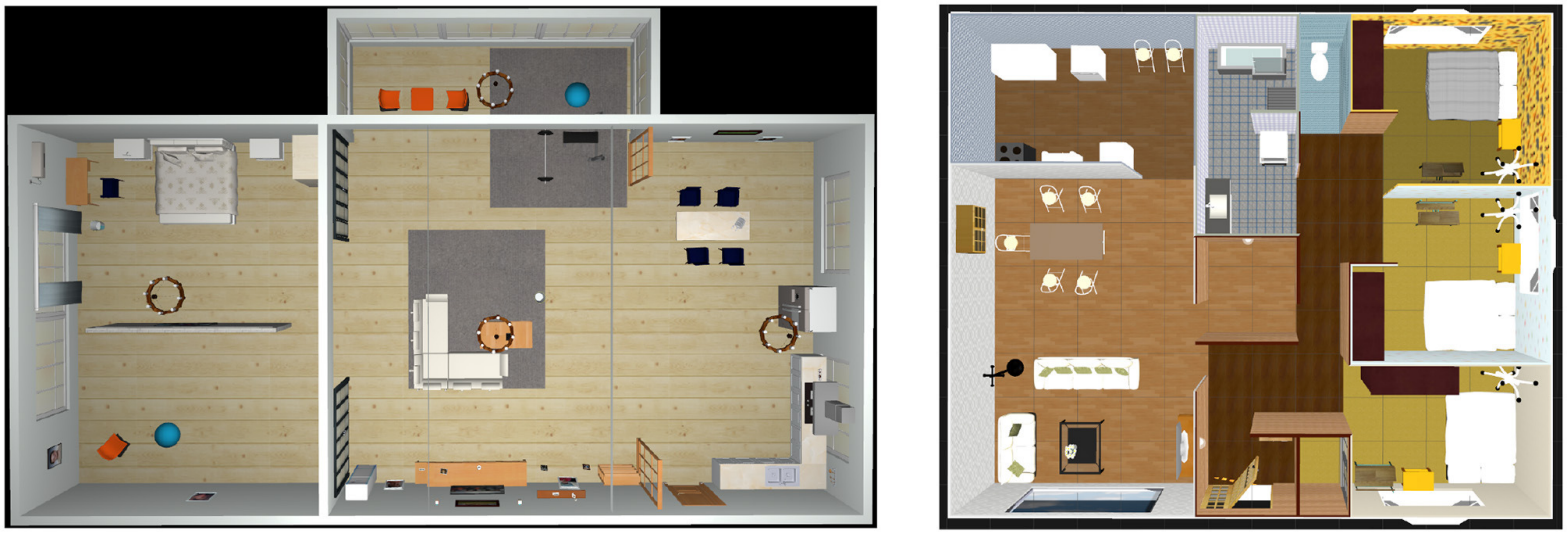
Bird view of experiment environment. **(Left)** Environment 1 and **(Right)** Environment 2.

The experiments were conducted using a robot operating system (ROS) (Quigley et al., 2009)/Gazebo environment. Two home environments with different wallpaper designs and layouts are shown in Figure 5. Environment 1 is an AWS small house^1^ environment with a size of ~11 × 19 m. Environment 2 was created using the interior design application SeetHome 3D and is ~10 × 12 m in size.

The robot is a Turtlebot3 Waffle Pi equipped with a 360° LiDAR sensor with a detection distance of 3.5 m and an RGB camera with a horizontal field of view (FOV) of 1.085 at a height of 1 m. Turtlebot3 is a two-wheeled robot with wheels driven by servomotors that can obtain odometry data calculated from the rotation data of each wheel. An environmental occupancy grid map is created for each environment in advance.

#### 4.1.1 Training

The training data for the RSSM and MRSSM modules consist of a sequence of two types of information: the observed image *o*_*t*_ captured by the RGB camera and the pose *x*_*t*_ estimated by MCL. The ROS AMCL (Adaptive Monte Carlo localization) package was used to implement MCL. The image was resized to 256 × 256 pixels after clipping the 480 × 640 pixel data obtained from the RGB camera to 480 × 480 pixels. The pose is that of the particle with the largest cumulative weight of MCL during the environmental exploration, and the ROS AMCL package publishes it. In the default settings of the ROS AMCL package, a translational movement of at least 0.25 m is required before performing a filter update. Additionally, the maximum speed of the Turtlebot3 is 0.26 m/s. Therefore, during the robot's translational movement, the particles are updated at ~1.3 Hz. The robot explored the environment by following human operations, and the training data were sampled at 1 Hz to approximately match the order of intervals between MCL particle updates. In other words, the state transition model of the RSSM or MRSSM model trained with these data is trained to predict the state one second later. We collected learning data for 365 and 186 min in Environments 1 and 2, respectively. The episodes in the training data do not include a kidnapped robot problem. We trained each model with the training data from each environment. This approach means that we independently conducted four trainings, corresponding to the combination of two models and two environments. Network training was performed under the following conditions: Using Adam, we trained for 3 k iterations at a learning rate of 10^−3^. The number of dimensions of the latent variables *h* and *s* was set to 200 and 30, respectively.

#### 4.1.2 Evaluation

**Figure 6:**
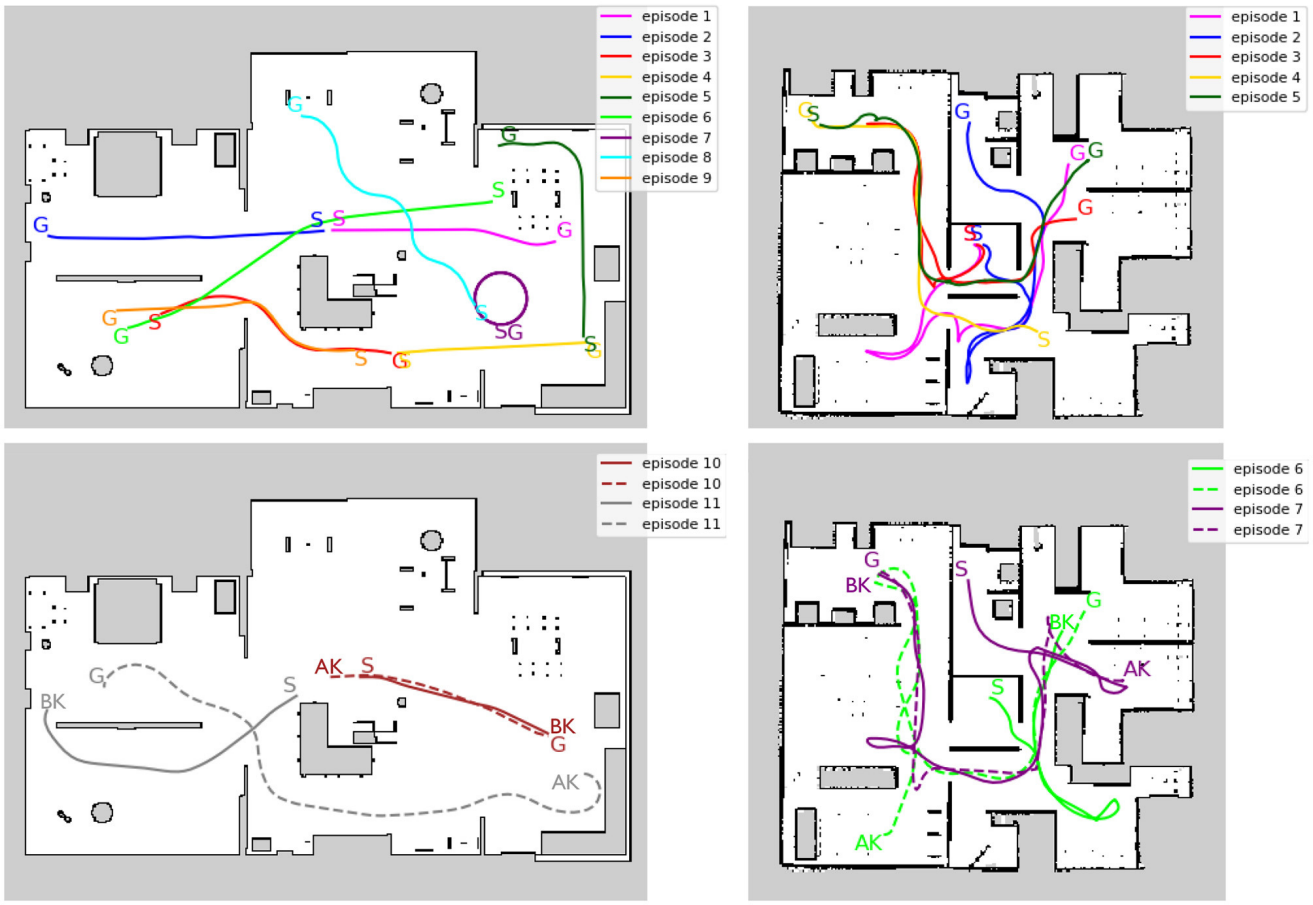
Trajectory of evaluation episodes written on the occupancy grid map. **(Left)** Environment1. **(Right)** Environment2. The top row shows that the episodes do not include kidnapping. The bottom row shows the episodes that include kidnapping. S, G, AK, and BK denote the starting point, the goal point, and the point before and after the kidnapping occurred in each episode, respectively. Solid and dotted lines indicate the trajectory before and after the kidnapping, respectively. The occupied grid map shows white areas as free, black areas as occupied, and gray areas as unobserved.

Separate from the training data, short exploration episodes were created for evaluation. Figure 6 illustrates the trajectory of an evaluation episode on an occupancy grid map. In Environment 1, episodes 1 and 2 start from the center (origin) of the environment, while episodes 3–9 start from different locations to evaluate global localization performance. Episodes 10 and 11 are designed specifically to address the kidnapped robot problem. In Environment 2, episodes 1 to 3 start from the center, episodes 4 and 5 start from different locations, and episodes 6 and 7 are designed for the kidnapped robot problem.

### 4.2 Result

#### 4.2.1 Localization performance

We evaluated the global self-localization performance of the trained model using the evaluation episodes. For each episode, we compared the performance of MCL, EMCL^2^, Models 1 and 2. Initially, the robot was assumed to have no information regarding its position. In other words, particles that approximate the initial self-pose are sampled from a uniform distribution in the environment. A total of 20 trials were conducted for each episode. In this study, the number of particles is fixed at 2,000.

Self-localization performance was evaluated using the root mean square error (RMSE), as shown in Equation (10). Here, *x*_*t*_, *y*_*t*_, θ_*t*_ is the pose of the particle with the largest cumulative weight value among the particles that the model infers. $\bar{x_{t}},\bar{y_{t}},\bar{\theta_{t}}$ is the ground truth pose.


(10) $$\begin{array}{ll} e_{t} & =\sqrt{{\left(\bar{x_{t}}-x_{t}\right)}^{2}+{\left(\bar{y_{t}}-y_{t}\right)}^{2}+{\left(\cos\bar{\theta_{t}}-\cos\theta_{t}\right)}^{2}+\left(\sin\bar{\theta_{t}}-\sin\theta_{t}\right)} \end{array}$$

Table 1 lists the average *e*_*t*_ values of 20 trials for each episode in both environments. The error is the standard deviation that represents the variation in each trial. In episodes without the kidnapped robot problem, Model 1 outperformed other methods in episodes 1 and 2 of Environment 1 and episodes 1 to 3 of Environment 2, which started from the center of the environment, while in other episodes, EMCL and Model 2 showed the best values depending on the episode. Model 2 showed the best values for episodes 10 and 11 in environment 1 and episodes 6 and 7 in environment 2, including the kidnapped robot problem. There were no episodes in which MCL exhibited the best performance.

**Table 1:** Results of RMSE in evaluation episodes.

**Type**	**Episodes**	**MCL**	**EMCL**	**Model 1**	**Model 2**
**Environment 1**					
No kidnapping	1	1.55 ± 1.11	0.23 ± 0.50	0.21 **±** **0.01**	0.92 ± 0.45
	2	1.23 ± 0.73	0.10 ± 0.00	0.09 **±** **0.01**	1.04 ± 0.62
	3	1.62 ± 1.21	0.82 **±** **1.39**	1.28 ± 0.66	1.45 ± 1.30
	4	1.48 ± 1.54	0.13 **±** **0.06**	1.88 ± 0.43	1.25 ± 0.42
	5	3.24 ± 1.53	3.29 ± 2.21	3.68 ± 0.96	2.67 **±** **0.89**
	6	1.34 ± 0.91	2.15 ± 1.83	2.51 ± 0.61	0.92 **±** **0.26**
	7	1.26 ± 0.86	1.93 ± 2.42	2.71 ± 0.94	0.30 **±** **0.11**
	8	1.06 ± 0.75	1.65 ± 2.18	2.18 ± 0.33	0.62 **±** **0.20**
	9	1.21 ± 1.12	0.49 ± 1.14	0.71 ± 0.23	0.30 **±** **0.14**
Kidnapping	10	2.46 ± 0.90	2.12 ± 0.91	1.10 ± 0.02	1.01 **±** **0.26**
	11	3.79 ± 0.39	3.76 ± 0.43	3.11 ± 0.02	1.14 **±** **0.13**
**Environment 2**					
No kidnapping	1	1.26 ± 1.08	0.78 ± 1.13	0.26 **±** **0.05**	0.37 ± 0.04
	2	1.16 ± 1.01	0.53 ± 0.91	0.22 **±** **0.05**	0.26 ± 0.03
	3	0.66 ± 0.80	0.49 ± 0.77	0.20 **±** **0.02**	0.41 ± 0.05
	4	1.60 ± 1.33	0.35 **±** **0.76**	2.11 ± 0.66	0.55 ± 0.06
	5	1.01 ± 1.19	0.38 **±** **0.74**	1.73 ± 0.66	0.42 ± 0.14
Kidnapping	6	2.16 ± 0.44	2.13 ± 0.47	1.57 ± 0.07	0.40 **±** **0.04**
	7	1.57 ± 0.33	1.31 ± 0.41	1.57 ± 0.19	0.31 **±** **0.02**

MCL, EMCL, Model 1, and Model 2. Bold and u means the best value. U means the second best value.

**Figure 7:**
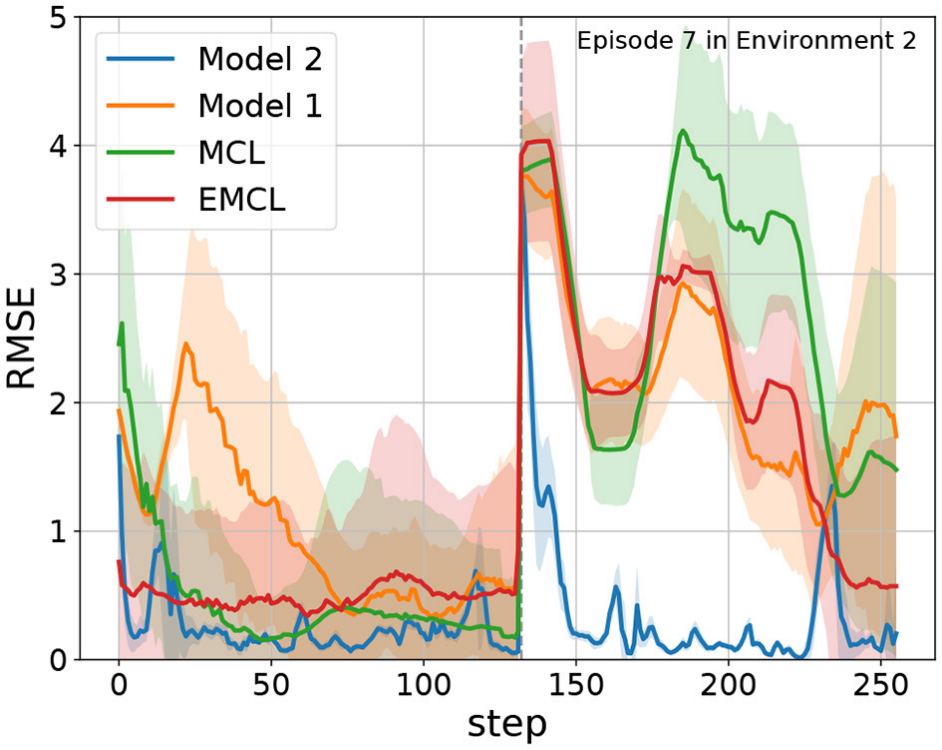
The RMSE of Episode 7 in Environment 2. The blue, orange, green, and red lines represent Model 2, Model 1, MCL, and EMCL, respectively. The dashed line indicates kidnapping at step 132.

To evaluate recovery performance from the kidnapped robot problem, Table 2 displays the average number of steps required for recovery and the success rates across 20 trials. A dash indicates that there was no success, and the values in parentheses represent the success rates. Recovery is defined as successful if the difference between the true pose and the estimated pose is within 0.5 meters and 0.1 radians at least once. Throughout all episodes, Model 2 recovered quickly and maintained a high success rate. As a specific example of the history of RSME, we show an example of episode 7 in Environment 2, which includes the kidnapped robot problem. The trajectory of this episode is indicated by the purple color in the bottom right figure of Figure 6. S, BK, AK, and G represent the start point, the point before kidnapping, the point after kidnapping, and the goal point, respectively. Figure 7 shows the trend of *e*_*t*_. Step 132 corresponds to the timing of the kidnapped robot problem. When kidnapping occurred, the RMSE of all models increased; however, Model 2 quickly reduced the error and recovered quickly from the kidnapped robot problem.

**Table 2:** The average number of steps taken to recover and success rate in kidnapping episodes.

**Environment**	**Episodes**	**MCL**	**EMCL**	**Model 1**	**Model 2**
1	10	–	–	–	18.4 ± 4.2 (0.45)
1	11	–	–	–	33.2 ± 2.4 (1.00)
2	6	–	132 (0.05)	–	36.1 ± 8.1 (1.00)
2	7	100 (0.05)	101.2 ± 0.4 (0.65)	101.0 ± 19.0 (0.45)	17.7 ± 3.0 (1.00)

#### 4.2.2 Representation of latent variables

Place cells have a high firing rate at specific locations in the environment and have been discovered in the rat CA3. In this study, we confirmed the representation obtained for the latent variable *h*_*t*_ corresponding to the region of CA1, CA3, and DG (Model 1) or CA3 and DG (Model 2). This finding indicates that each of the 200 dimensions of the latent variable corresponds to a cell in the corresponding region.

**Figure 8:**
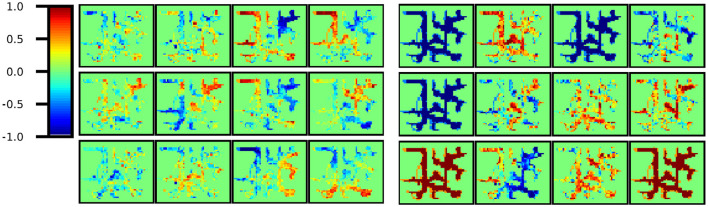
Part of *h*_*t*_ cells. **(Left)** All cells obtained in Model 1 yielded different firing rates in different locations. In other words, location-distinguishable cells were obtained. **(Right)** However, in Model 2, some cells remain constant regardless of location and are not location-distinguishable cells. In other words, a sparse representation was obtained in total.

First, using the trained model, the latent variable *h*_*t*_ was inferred at each step of the training data, and all latent variables were associated with the pose estimated by MCL. Then, The area of Environment 2 (10 × 12 m) was divided into 0.25 m square bins, and Figure 8 shows a plot of the average values in each bin for a part of cells. All 200 cells are shown in Figure S1. All the cells obtained with Model 1 had different firing rates at different positions. In contrast, in Model 2, some cells fired at a constant rate regardless of location(i.e., Indistinguishable location cells). In other words, a sparse representation was obtained overall. This sparse representation obtained in CA3 is consistent with the findings of neuroscience.

## 5 Discussion

In this section, we discuss how our findings, particularly the results of Model 2, relate to neuroscience, specifically to the structure and function of the hippocampus.

The results of the localization experiment suggest that Model 1 tends to be more influenced by preconceived beliefs than Model 2. In global self-localization tasks, episodes starting near the center of the environment tend to perform better with Model 1. The reason is thought to be that, in the implementation, the initial value of *h*_0_ is set to correspond to the center of the environment. Moreover, in episodes including the kidnapped robot problem, Model 2 demonstrated better performance than Model 1. These results indicate that Model 1 is more susceptible to inheriting the state from a previous time compared to Model 2 (i.e., tends to be influenced by preconceived beliefs). Moreover, in comparison with EMCL, Model2 demonstrated superior performance, especially in scenarios involving the kidnapped robot problem. EMCL performs expansion resetting when the integral of the weighted likelihood of observations falls below a threshold. This method expands the current belief distribution in the pose space to search for solutions and assumes the inductive bias that the robot remains nearby even after a kidnapping. However, in environments where similar observations can be made at multiple locations, there is a bias toward solutions close to the current belief distribution in pose space, which can lead to convergence to local optima.

From the perspective of state-space models, the main difference between Models 1 and 2 lies in whether the information about robot pose *x*_*t*_ is given as input (action) or output (observation) to the state-space model. As many state-space models assume that self-behavior directly and predictably impacts the environmental state, in RSSM, actions deterministically influence the state at the next moment through the dynamics of the state-space learned by GRU. In Model 1, the input *x*_*t*_ deterministically affects the subsequent state *h*_*t*+1_, whereas in Model 2, the observation *x*_*t*_ influences the state *h*_*t*_ through the inference model along with image *o*_*t*_ by using a multimodal VAE. The multimodal VAE is capable of learning robust representations that are shared across multiple modalities, even when some modalities are partially available. Therefore, in Model 2, even if the self-location suddenly becomes incorrect (i.e., teleportation), the robot can quickly recover from the kidnapped robot problem.

This scenario raises the question of when there is a difference between the allocentric self-location belief and the images currently being seen, which is concluded to be correct. One possibility is the theory that CA3's recurrent collateral pathway learns an attractor network from past experiences and draws it in by obtaining a sparse representation (Kesner and Rolls, 2015). We showed that the variable *h*_*t*_, corresponding to CA3 and DG, distinguished locations using fewer dimensions (cells) and found that its sparse representation (Figure 8) was consistent with neuroscientific findings (Jezek et al., 2011). From the perspective of predictive coding, considering the function of top-down predictions, the region integrating information necessitates dense information to forecast lower-level complex multimodal inputs, thereby complicating the acquisition of sparse representations (*e.g*., Model 1). However, structuring the model to separate the variable updated recursively with GRU from the variable that integrates information automatically enables the acquisition of sparse representations, which automatically facilitates adaptation to unprecedented teleportation events by leveraging past experiences (*e.g*., Model 2).

The discussion thus far indicates that the functions and structure of the hippocampal formation are closely related. In Model 2, the architecture was designed such that the multimodal VAE and the GRU correspond, respectively, to the temporo-ammonic pathway of CA1 and the recurrent collateral pathway of CA3, characteristic hippocampus structures. Despite the absence of manual design of relations between variables, the self-supervised method enables the GRU hidden layer to reproduce features known as representations of CA3, such as place cell and sparsity. It also demonstrates the capability of adapting self-localization functions, even in the kidnapped robot problem. This observation indicates that the structure of the hippocampal formation is a primary factor affecting its recognized features and functions.

## 6 Conclusion

In this study, we created a computational model of spatial cognition inspired by hippocampal formation by integrating RSSM or MRSSM and MCL, which are two types of probabilistic generative models, using Neuro-SERKET. We compared the localization performance of two hypothetical integrated models with different granularities of hippocampal modeling. We found that the calculation model that distinguishes the CA1 and CA3 of the hippocampus has better self-localization performance when teleportation occurs. We also showed that a sparse representation is obtained for random variables corresponding to CA3 in the computational model, which is thought to be due to the structural factors in the computational model. These results suggest that machine learning models incorporating insights into the brain's structural information offer robustness in situations not anticipated during learning or design phases, such as the kidnapped robot problem.

The limitations of this study were as follows: The allocentric representation is assumed to be the pose on the occupancy grid map and is given in advance. When an engineer provides a map to a computation model, its format and the relationship between the sensors and map information are fixed based on prior knowledge.

Therefore, as a future challenge, it will be necessary to construct a representation of the environment from the robot's sensor information without the aid of map information, that is, to perform SLAM. Additionally, it may be possible to generate behavior from a robust representation. In this study, we showed that beliefs were appropriately updated in the kidnapped robot problem, but no actions were generated. The ability to estimate one's state robustly, even when a situation changes dynamically, is necessary for robust navigation in such a dynamic environment.

## Data availability statement

The raw data supporting the conclusions of this article will be made available by the authors, without undue reservation.

## Author contributions

TN: Writing—original draft, Writing—review & editing, Formal analysis. SO: Writing—review & editing, Writing—original draft, Formal analysis. AT: Writing—review & editing, Writing—original draft, Conceptualization, Funding acquisition. KM: Writing—review & editing, Writing—original draft, Software. LE: Writing—review & editing, Writing—original draft, Software. TT: Writing—review & editing, Writing—original draft. HY: Writing—review & editing, Writing—original draft, Conceptualization, Funding acquisition.

## Footnotes

^1^ https://github.com/aws-robotics/aws-robomaker-small-house-world

^2^ https://github.com/ryuichiueda/emcl2
