# Supplementary material for: Hippocampal formation-inspired global self-localization: quick recovery from the kidnapped robot problem from an egocentric perspective
Source: Front Comput Neurosci. 2024 Jul 18;18:1398851. doi: 10.3389/fncom.2024.1398851 (PMC11291353; doi:10.3389/fncom.2024.1398851)
Supplement: Supplementary file 1 [file Data_Sheet_1.pdf]

Supplementary Material

1 DERIVATION OF BAYES FILTER IN PROPOSED MODELS

Belief represents the conditional probability distribution of the latent variables within the model, as shown in the following Equation(S1).

$$
\begin{aligned}
&\mathrm{Bel}(t) \\
&\triangleq p(x_{1:t}, h_{1:t}, s_{1:t}|o_{1:t}, z_{1:t}, u_{1:t}) \\
&\propto p(o_t, z_t|x_{1:t}, h_{1:t}, s_{1:t}, o_{1:t-1}, z_{1:t-1}, u_{1:t}) \\
&\quad\; p(x_{1:t}, h_{1:t}, s_{1:t}|o_{1:t-1}, z_{1:t-1}, u_{1:t}) && \text{(Bayes' theorem)} \\
&= p(o_t, z_t|x_{1:t}, h_{1:t}, s_{1:t}, o_{1:t-1}, z_{1:t-1}, u_{1:t}) \\
&\quad\; p(x_t, h_t, s_t|x_{1:t-1}, h_{1:t-1}, s_{1:t-1}, o_{1:t-1}, z_{1:t-1}, u_{1:t}) \\
&\quad\; p(x_{1:t-1}, h_{1:t-1}, s_{1:t-1}|o_{1:t-1}, z_{1:t-1}, u_{1:t}) \\
&= p(o_t, z_t|x_t, h_t, s_t) \\
&\quad\; p(x_t, h_t, s_t|x_{t-1}, h_{t-1}, s_{t-1}, u_t) && \text{(Markov property)} \\
&\quad\; p(x_{1:t-1}, h_{1:t-1}, s_{1:t-1}|o_{1:t-1}, z_{1:t-1}, u_{1:t-1}) \\
&= \underbrace{p(o_t, z_t|x_t, h_t, s_t)}_{\text{measurement update}}\underbrace{p(x_t, h_t, s_t|x_{t-1}, h_{t-1}, s_{t-1}, u_t)}_{\text{control update/prediction}}\mathrm{Bel}(t-1)
\end{aligned} \tag{S1}
$$

First, Bayes' theorem is used to factorize into the likelihood of observations at time t and prior distribution. Then, the prior distribution is factorized into the prediction of latent variables at time t and the belief at previous time $t-1$ using the probability multiplication theorem. Finally, we considered the dependence of the variables assumed during modeling and removed random variables with no dependence on the conditions. In this case, since the Markov property is assumed, the behavioral model only appears as random variables at times t and $t-1$ and is in the form of a sequential equation.

A state-space model using a Bayes filter alternately updates beliefs based on input (behavior) and output(observation). The former is called a control update or prediction, and the latter is called a measurement update. Equation (S1) can be further expanded by considering the dependence of variables in each model. In Model 1, measurement update is

$$p(o_t, z_t|x_t, h_t, s_t) = p(z_t|x_t)p(o_t|s_t),$$

and the behavior update is

$$p(x_t, h_t, s_t|x_{t-1}, h_{t-1}, s_{t-1}, u_t) = p(s_t|h_t)p(x_t|h_t)f^{GRU}(h_{t-1}, s_{t-1}, x_{t-1})p(x_t|x_{t-1}, u_t).$$

Therefore, in Model 1, Equation (S1) can be rewritten as the following Equation (S2).

$$\begin{aligned} &\mathrm{Bel}(t) \\ &\propto p(o_t, z_t|x_t, h_t, s_t)p(x_t, h_t, s_t|x_{t-1}, h_{t-1}, s_{t-1}, u_t)\mathrm{Bel}(t-1) \\ &= p(z_t|x_t)\underbrace{p(o_t|s_t)p(s_t|h_t)}_{q(s_t|h_t,o_t)}p(x_t|h_t)f^{GRU}(h_{t-1}, s_{t-1}, x_{t-1})p(x_t|x_{t-1}, u_t)\mathrm{Bel}(t-1) \end{aligned} \tag{S2}$$

On the other hand, in Model 2, the measurement update is

$$p(o_t, z_t|x_t, h_t, s_t) = p(z_t|x_t)p(o_t|h_t, s_t),$$

and the behavior update is

$$p(x_t, h_t, s_t|x_{t-1}, h_{t-1}, s_{t-1}, u_t) = p(s_t|h_t)p(x_t|h_t, s_t)f^{GRU}(h_{t-1}, s_{t-1})p(x_t|x_{t-1}, u_t).$$

Therefore, in Model 2, Equation (S1) is rewritten as the following Equation (S3).

$$\begin{aligned} &\mathrm{Bel}(t) \\ &\propto p(o_t, z_t|x_t, h_t, s_t)p(x_t, h_t, s_t|x_{t-1}, h_{t-1}, s_{t-1}, u_t)\mathrm{Bel}(t-1) \\ &= p(z_t|x_t)\underbrace{p(o_t|h_t, s_t)p(s_t|h_t)}_{q(s_t|h_t,o_t)}p(x_t|h_t, s_t)f^{GRU}(h_{t-1}, s_{t-1})p(x_t|x_{t-1}, u_t)\mathrm{Bel}(t-1) \end{aligned} \tag{S3}$$

2 LATENT VARIABLE REPRESENTATION

Figure S1 shows the average firing rates for all 200 dimensions of h_t. Since h_t uses the activation function tanh, it takes a value between -1 and 1. The 10 m × 12 m area of Environment 2 was divided into 0.25 m square bins, and the latent variables at each location were inferred. The data were time-series data obtained by exploring the environment for 186 min, which was also used for model training. As each bin was visited multiple times, the average firing rate of h_t is displayed.

3 ARCHITECTURE

The architecture of Model2 is shown in Table S1. The Image Encoder uses CNNs to obtain a 1024-dimensional embedding vector from a 3-channel RGB image. The Image Decoder uses CNNs to generate RGB images from latent variables. The Pose Encoder uses five layers of fully connected layers to obtain a 1024-dimensional embedding vector from 4-dimensional pose information. Note that the pose (x_t, y_t, θ_t) is converted to $(x_t, y_t, \cos\theta_t, \sin\theta_t)$ for input into the neural network. The Pose Decoder uses three fully connected layers to obtain the mean and variance of the pose distribution approximated by a multivariate Gaussian distribution from latent variables. A type of RNN, the GRU, was used in the transition model to learn dynamics in the state space. Adding a dummy value of zero, due to implementation issues, which should correspond to the dimension for the pose, makes the total 34 dimensions. The dimensions of both the hidden layer and the input of the GRU are set to 200. Probabilistic latent variables of the input information are first projected to 200 dimensions using a fully connected layer before being input into the GRU. FC1 is

a model within the framework of VAEs that infers the probabilistic distribution of latent variables modeled as a multivariate Gaussian distribution, outputting not only the mean but also the standard deviation of the distribution.

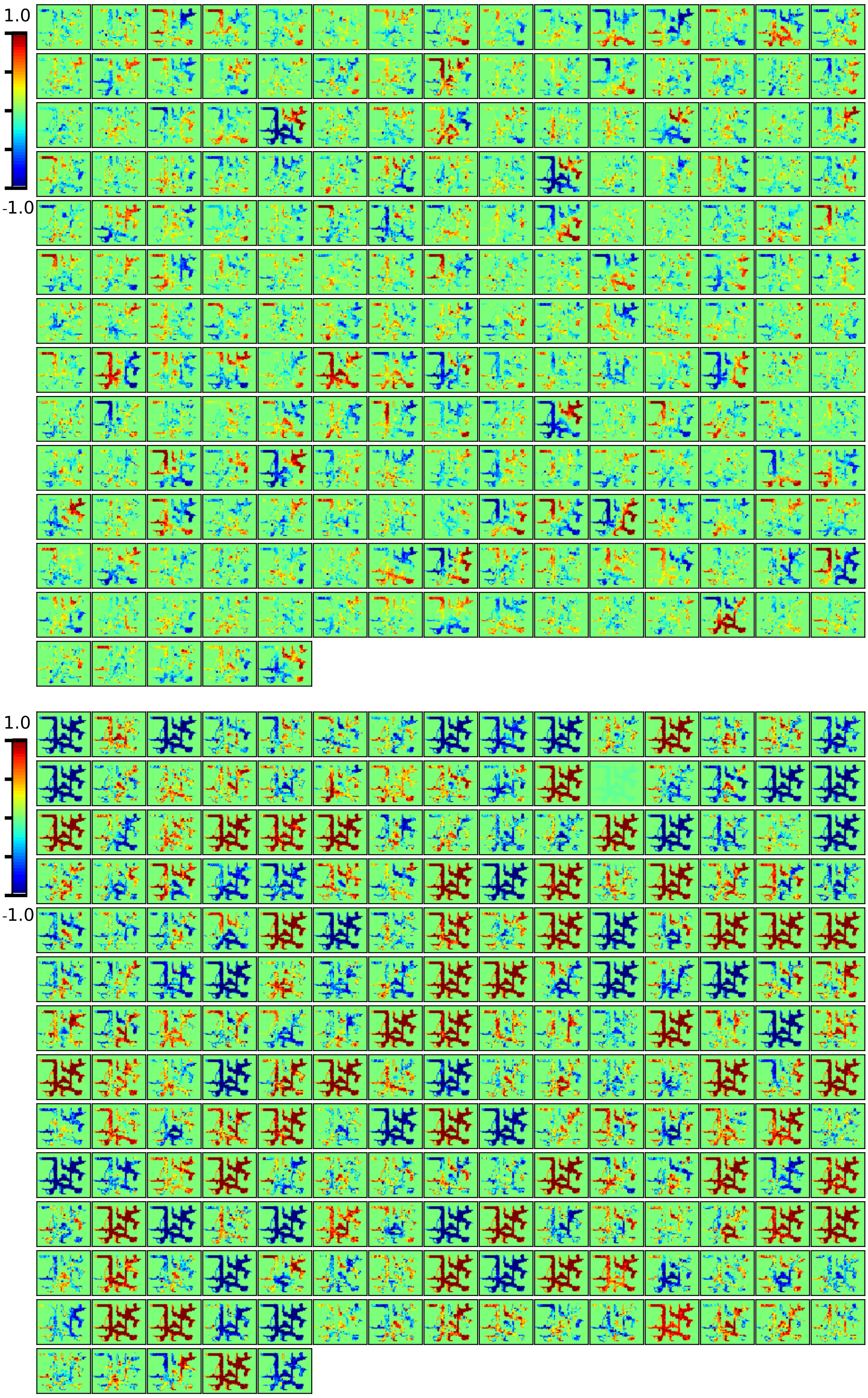

Figure S1. Average firing rate of h_t. The value of h_t has values from -1 to 1. (Top) In Model 1, all 200 cells are location-distinguishable. (Bottom) On the other hand, in Model 2, some cells have the same value in all locations and do not change. The number of location-distinguishable cells is limited, and the representation is sparse.

Table S1. Model 2 architecture.

Image Encoder $q(e_t^{(o)}|o_t)$
Input $o_t \in \mathbb{R}^{3\times256\times256}$, Output $e_t^{mean} \times e_t^{std.} \in \mathbb{R}^{2048}$
Conv.: 4×4 kernel, 8 out-channel, 2×2 stride, Batch norm 2d & ReLU
Conv.: 4×4 kernel, 16 out-channel, 2×2 stride, Batch norm 2d & ReLU
Conv.: 4×4 kernel, 32 out-channel, 2×2 stride, Batch norm 2d & ReLU
Conv.: 4×4 kernel, 64 out-channel, 2×2 stride, Batch norm 2d & ReLU
Conv.: 4×4 kernel, 128 out-channel, 2×2 stride, Batch norm 2d & ReLU
Conv.: 4×4 kernel, 256 out-channel, 2×2 stride, Batch norm 2d & ReLU
Reshape 1024
Full connected layer: 1024 in-features, 1024 out-features & ELU
Full connected layer: 1024 in-features, 2048 out-features

Image Decoder $p(o_t|h_t, s_t)$
Input $h_t \in \mathbb{R}^{200}$, $s_t \in \mathbb{R}^{30}$, Output $o_t \in \mathbb{R}^{3\times256\times256}$
Full connected layer: 30 in-features, 1024 out-features
Deconv.: 6×6 kernel, 256 out-channel, 2×2 stride, Batch norm 2d & ReLU
Deconv.: 4×4 kernel, 128 out-channel, 2×2 stride, Batch norm 2d & ReLU
Deconv.: 4×4 kernel, 68 out-channel, 2×2 stride, Batch norm 2d & ReLU
Deconv.: 4×4 kernel, 32 out-channel, 2×2 stride, Batch norm 2d & ReLU
Deconv.: 4×4 kernel, 16 out-channel, 2×2 stride, Batch norm 2d & ReLU
Deconv.: 6×6 kernel, 3 out-channel, 2×2 stride

Pose Encoder $q(e_t^{(x)}|x_t)$
Input $x_t \in \mathbb{R}^4$, Output $e_t^{mean} \times e_t^{std.} \in \mathbb{R}^{2048}$
Full connected layer: 4 in-features, 1024 out-features & ELU
Full connected layer: 1024 in-features, 1024 out-features & ELU
Full connected layer: 1024 in-features, 1024 out-features & ELU
Full connected layer: 1024 in-features, 1024 out-features & ELU
Full connected layer: 1024 in-features, 2048 out-features

Pose Decoder $q(x_t|h_t, s_t)$
Input $h_t \in \mathbb{R}^{200}$, $s_t \in \mathbb{R}^{30}$, Output $x_t^{mean} \times x_t^{std.} \in \mathbb{R}^8$
Full connected layer: 230 in-features, 128 out-features & ELU
Full connected layer: 128 in-features, 128 out-features & ELU
Full connected layer: 128 in-features, 8 out-features

Transition model(GRU)
Input $h_{t-1} \in \mathbb{R}^{200}$, $s_{t-1} \in \mathbb{R}^{30}$, Output $h_t \in \mathbb{R}^{200}$
Full connected layer: 34 in-features, 200 out-features & ReLU
GRU: 200 hidden-size, 200 input-size

Posterior(FC1) $p(s_t^q|h_t, e_t)$
Input $h_t \in \mathbb{R}^{200}$, $e_t \in \mathbb{R}^{1024}$, Output $s_t^{mean} \times s_t^{std.} \in \mathbb{R}^{60}$
Full connected layer: 1224 in-features, 200 out-features & ReLU
Full connected layer: 200 in-features, 60 out-features

Prior(FC2) $p(s_t^p|h_t)$
Input $h_t \in \mathbb{R}^{200}$, Output $s_t^{mean} \times s_t^{std.} \in \mathbb{R}^{60}$
Full connected layer: 200 in-features, 200 out-features & ReLU
Full connected layer: 200 in-features, 60 out-features
